# Emergency Department and Urgent Care Medical Malpractice Claims 2001–15

**DOI:** 10.5811/westjem.2020.9.48845

**Published:** 2021-02-15

**Authors:** Kelly E. Wong, P. Divya Parikh, Kwon C. Miller, Mark R. Zonfrillo

**Affiliations:** * Alpert Medical School of Brown University, Department of Emergency Medicine, Providence, Rhode Island; † Medical Professional Liability Association, Department of Research and Education, Rockville, Maryland; ‡ Medical Professional Liability Association, Department of Research Database Management, Rockville, Maryland; § Alpert Medical School of Brown University, Departments of Emergency Medicine and Pediatrics, Providence, Rhode Island

## Abstract

**Introduction:**

This study reviews malpractice, also called medical professional liability (MPL), claims involving adult patients cared for in emergency departments (ED) and urgent care settings.

**Methods:**

We conducted a retrospective review of closed MPL claims of adults over 18 years, from the Medical Professional Liability Association’s Data Sharing Project database from 2001–2015, identifying 6,779 closed claims. Data included the total amount, origin, top medical specialties named, chief medical factors, top medical conditions, severity of injury, resolution, average indemnity, and defense costs of closed claims.

**Results:**

Of 6,779 closed claims, 65.9% were dropped, withdrawn, or dismissed. Another 22.8% of claims settled for an average indemnity of $297,709. Of the 515 (7.6%) cases that went to trial, juries returned verdicts for the defendant in 92.6% of cases (477/515). The remaining 7.4% of cases (38/515) were jury verdicts for the plaintiff, with an average indemnity of $816,909. The most common resulting medical condition cited in paid claims was cardiac or cardiorespiratory arrest (10.4%). Error in diagnosis was the most common chief medical error cited in closed claims. Death was the most common level of severity listed in closed (38.5%) and paid (42.8%) claims. Claims reporting major permanent injury had the highest paid-to-closed ratio, and those reporting grave injury had the highest average indemnity of $686,239.

**Conclusion:**

This retrospective review updates the body of knowledge surrounding medical professional liability and represents the most recent analysis of claims in emergency medicine. As the majority of emergency providers will be named in a MPL claim during their career, it is essential to have a better understanding of the most common factors resulting in MPL claims.

## INTRODUCTION

Among the challenges in emergency departments (ED) is providing quality care to patients with high-risk diagnoses under the pressures of limited information and increasing demands on time. This complex environment inherently lends itself to potential medical errors and possible resulting litigation. The threat of a malpractice lawsuit partially drives physicians’ clinical decision-making in such environments. A survey performed by the Harvard School of Public Health and Columbia Law School found that 93% of physicians in high-risk specialties change their clinical decision-making due to concern of a malpractice suit, a behavior commonly referred to as “defensive medicine.”^1^

A prior study by Brown et al. in 2010 examined 11,529 closed medical professional liability (MPL) claims originating from EDs for adult patients between 1987–2007 using a database from the Physician Insurers Association of America (the former name of the MPL Association).^2^ The changing landscape of MPL due to tort reform, fluctuations in malpractice insurance premiums, and regulatory interference underscores the need for a more contemporary analysis of the MPL data. This study reviewed MPL claims involving adult patients (over 18 years old) cared for in ED and urgent care settings and provides an update of characteristics in closed MPL claims from 2001–2015.

## METHODS

In this retrospective review of closed adult MPL claims reported to the Data Sharing Project (DSP) of the MPL Association during a 15-year period (2001–2015), we reviewed 135,490 closed claims. The DSP is the largest independent database of MPL claims and lawsuits, comprised of aggregated and de-identified information from voluntarily participating member insurance companies. The MPL Association represents more than two-thirds of physicians in private practice.

We queried the DSP for MPL claims involving adult patients (older than 18 years) with claims arising from care received in a United States hospital-based ED or ambulatory urgent care center. Information obtained included the medical specialty involved, top resulting medical conditions, chief medical factor, and severity of resulting injury. We analyzed the outcomes of these claims (i.e., dropped, settled, judgment for plaintiff or defendant, etc.), as well as the amount of the award to the plaintiffs and the total defense fees. We summarized data using summary statistics. This study of de-identified data was not considered human subjects research by our institutional review board.

## RESULTS

Of 135,490 MPL claims and lawsuits closed between 2001–2015, 6,779 (5%) involved adult patients over 18 years old in a US hospital-based ED or ambulatory urgent care setting (Figure) (Table 1). The ED represented 5.2% of adult claims from all facilities, and urgent cares represented 0.9% of claims. Of hospital-based origins, the ED was the third most common origin (9.1%) for a claim, following operating rooms (40.7%) and inpatient rooms (15.8%).

Of the 6,779 total claims, 65.9% were dropped, withdrawn or dismissed. Another 22.8% were settled for an average indemnity of $297,709 and an average defense expense of $55,260. Of the 515 (7.6%) cases that went to trial, juries returned verdicts finding for the defendant in 92.6% of cases (477/515). The average defense cost for a verdict in favor of the defendant was $111,446. The remaining 7.4% of cases (38/515) where juries returned verdicts for the plaintiff had an average indemnity of $816,909 and an average defense expense of $159,716. There were 222 claims (3.3%) that resulted in alternative dispute resolution (ADR) or private contract. There were 30 claims (0.4%) with an unknown outcome (Table 2).

Emergency physicians were the primary specialty named in 33.5% of the 6,779 closed claims, followed by internal medicine (12.4%), family practice (9.6%), radiology (7.3%), and general surgery (7.1%).

**Population Health Research Capsule**

What do we already know about this issue?

Approximately 75% of emergency physicians will be named in a malpractice suit during their career and the average time to resolution is 16.7 months per claim.

What was the research question?

We sought to characterize closed claims involving adults originating from emergency departments or urgent care centers.

What was the major finding of the study?

A total of 65.9% of claims were dropped, 22.8% settled, 7.6% went to trial, 3.3% by private contract, and 0.4% unknown.

How does this improve population health?

Understanding the most common factors in recent closed malpractice claims provides important context to improve the care of adults treated in emergency settings.

For the 27.1% of closed MPL claims culminating in an indemnity payment (trial verdicts, verdicts for the plaintiff, ADR/contracts, and unknown), the resulting medical conditions most commonly cited were cardiac or cardiorespiratory arrest (9.1%), acute myocardial infarction (4.0%), aortic aneurysm (2.3%), pulmonary embolism (2.2%), and appendicitis (2.0%). Of these, acute myocardial infarction had the highest paid-to-closed ratio with 39% resulting in a payment. Claims for aortic aneurysms generated the highest average indemnity of $369,872 per claim (Table 3).

The chief medical errors cited in MPL closed claims, seen in Table 4, were errors in diagnosis (36.4%); no medical misadventure (19.2%); improper performance (17.7%); failure to supervise or monitor case (5.2%); and medication errors (3.4%).

As seen in Table 5, death was the most common severity of injury cited in closed adult MPL claims, listed in 38.5% of closed claims and 42.8% of paid claims. Claims reporting major permanent injury had the highest percent of paid-to-closed claims (38.3%), and grave injury had the highest average indemnity of $686,239. Emotional injury was the least likely severity of injury to be listed, comprising 0.9% of total claims, in addition to having the lowest paid-to-closed ratio at 11.7%.

## DISCUSSION

Making time-sensitive healthcare decisions for patients with myriad and complex conditions based on limited information is routine for emergency physicians (EP), but not without risk. Compared to other specialties where physicians may avoid caring for high-risk patients in order to mitigate medical liability, EPs are limited in their ability to choose their patient population.^1^ The American Medical Association found that 8.7% of respondents in emergency medicine faced a MPL claim in the prior year alone,^3^ and it is estimated that over 75% of EPs will be named in a malpractice suit by the end of their career.^4^

In the early 2000s, tort reform “intended to protect physicians who are practicing with incomplete information in high-intensity care settings”^5^ changed the definition of when physicians can be named in MPL and the manner in which those claims are resolved. For instance, the definition of malpractice in some states changed from “a deviation in standard care” to “gross negligence,”^5^ and over the same time period, nine states set a new cap on damages in MPL cases.^6^ Proponents of tort reform argue that these increased protections will result in decreased overall healthcare spending by assuaging physicians’ fears and changing their practice patterns; however, that has yet to be borne out in the literature. One analysis of MPL in three states (TX, GA, and SC) in the years immediately before and after tort reform observed no change in three proxies of defensive medicine practices: ordering computed tomography (CT) and magnetic resonance imaging (MRI); hospital admission;, and total charges for ED visits.^5^ Similarly, a retrospective study of EPs recently named in a malpractice suit compared to similar, unnamed peers found no difference in what they called care intensity (measured as admission rate or relative value units (RVU) per visit as a proxy for increased testing) or speed (measured as RVUs per hour or length of stay).^7^

Brown et al. examined closed MPL claims originating from EDs for adult patients from 1987–2007 using an overlapping but different data set. They found an average indemnity of $175,545 in settled claims and an average indemnity of $393,350 in verdicts found for the plaintiff. Of the 11,529 claims identified by their dataset, 64% were withdrawn, dropped, or dismissed with no payment paid to the plaintiff. Error in diagnosis was the most common category of error. Acute myocardial infarction was both the most common specific diagnosis cited and had the highest paid-to-closed ratio in their dataset, with 42% of all claims resulting in a payment.^2^

While there have been previous analyses of MPL, including Brown et al., the source data means no direct comparisons can be made. This retrospective review updates the body of knowledge surrounding medical liability and represents the most recent analysis of claims for adults treated in emergency or urgent care settings. Average indemnity of settled claims in our study (2001–2015) was $297,709, and average indemnity of claims where the plaintiff prevailed was $816,909. The majority of cases (92.6%) that proceeded to trial were found in favor of the defendant. The average defense fee when the verdict found for the defendant was $111,446. Even claims that were dropped, dismissed, or withdrawn had average defense fees of $25,996.

While we did not analyze trends over our study period, a review of all specialties during a similar time range (2004–2016) found an inflation-adjusted increase in all indemnity, with payments related to diagnosis-related errors increasing by 31.2%.^8^

Studies have estimated that EPs face an average time to resolution of 16.7 months for each open claim.^9^ This extended period of time has consequences for parties on either side of the claim. Plaintiffs and their families potentially face a delay in compensation, loss of work, and emotional repercussions of a protracted resolution. For physicians among all specialties, 50% of claims that ultimately resulted in no payment took more than one year to be resolved.^9^ Lost clinical time,^10^ in addition to defense fees and value of lost reputation,^11^ may negatively impact physicians, their careers, and their families.

Errors in diagnosis was the most common reason for a claim in this dataset, consistent with other adult^2^,^12^ and pediatric^13^,^14^ emergency medicine studies. Research focusing on the processes leading to an error in diagnosis in the ED identified four main categories: failure to order tests (58%); inadequate medical history and physical examination (42%); incorrect interpretation of tests (37%); and failure to request a consultation (33%).^15^

To avoid medical errors, EPs’ rapid access to most imaging and testing modalities without having to obtain prior authorization may contribute to costly and unnecessary utilization of resources. A survey of EPs’ most recent act of defensive medicine found that 63% of respondents ordered imaging (CT, MRI, or radiograph) that was not clinically indicated.^1^ Overtesting and overimaging is not without risk either; one MPL study of imaging in the ED found that 37% of diagnostic errors resulting in patient harm involved the misinterpretation of diagnostic testing, with plain radiographs being the most common at 52%.^15^

“No medical misadventure” was the second most common chief medical factor cited in claims. According to the MPL Association, “‘No medical misadventure” is a code used in the absence of a medical mishap. If a claim has no medical misadventure but is felt to have legal merit, there is an appropriate associated issue designated in the database. These can be problems with records, consent issues, laboratory issues or assault/battery, abandonment, etc.”^16^ Despite being the second most common cited reason for bringing a claim, only 3.3% of claims citing “no medical misadventure” resulted in a payout, and represented only 2.4% of total paid claims.

In our analysis, claims listing grave injury had more than double the average indemnity as paid claims listing death as the resulting injury ($686,239 vs $326,350). Death was the most common (38.5%) injury cited in all closed adult MPL claims, followed by minor temporary injury (15.1%) and major temporary injury (13.8%). A prior study examining MPL claim outcomes and time to resolution found that the more severe the injury listed in the claim, the longer the time to resolution.^9^ Among all specialties, 51% of claims with emotional injury only took at least six months to resolve. In 62% of claims listing death or permanent disability, the time to resolution was over one year, with 3% lasting longer than five years.^9^

Acute myocardial infarction was the diagnosis with the highest ratio of paid-to-closed claims. Chest pain continues to be one of the most common chief complaints in the hospital, representing 8–10 million visits per year,^17^ with acute ST-elevation myocardial infarctions (STEMI) representing an estimated 0.26% of ED visits.^17^ Risk stratification in this population may be aided by the introduction of high-sensitivity troponin and evidence-based decision tools; however, diagnosis of acute myocardial infarction is also affected by subjective interpretation of electrocardiograms that may vary between providers. The overall incidence of STEMIs seen in the ED has been decreasing in recent years. Both the push to improve time to reperfusion and the pre-hospital recognition of STEMIs may have contributed to this decrease, allowing patients to bypass EDs and present directly to catheterization labs. Ward et al. speculated that atypical presenting STEMIs that are more difficult to diagnose and treat may still present to the ED, while classically presenting STEMIs are more likely to proceed directly to the catheterization lab.^18^

Emergency medicine was the most commonly named specialty in our study, followed by internal medicine, family practice, radiology, and general surgery. EPs might view requesting a consult from another specialty as a way of mitigating risk. For example, a review of MPL involving point-of-care ultrasound found that 40% of those imaging studies were performed by radiology, even though both the study and its interpretation were within EPs’ scope of practice.^19^ A consulting physician-patient relationship must occur through “an overt or implied agreement to participate in a patient’s care, or by reviewing specific tests or studies for the purpose of diagnosis and treatment.”^20^ The case law surrounding shared liability underscores the challenge of delineating when a formal consultation has been made and highlights various occasions when EPs incorrectly presume that a formal consult (and therefore shared liability) was established.

It is our hope that these findings based on these MPL data may help to inform emergency providers about risks and outcomes, and may provide important context to improving the care of adults treated in emergency or urgent care settings.

## LIMITATIONS

This study has several limitations. While the DSP is the largest independent database of MPL claims and lawsuits, it does not capture all closed claims during the study period and may not be representative. In addition, because DSP data were in aggregate to ensure confidentiality, we were not able to obtain information about individual cases or trend claim-specific data over time from 2001–2015. Prior work on EP demographics has suggested that total number of years in practice and total visits seen were associated with increased risk of MPL;^21^ similarly, due to the aggregate data, we did not analyze demographics of individual physicians in this study. Additionally, average monetary values did not account for inflation rates, and were averaged over the 15-year period. We were unable to differentiate between types of aortic aneurysm in resulting medical condition, and this category comprises thoracic, abdominal, and thoracoabdominal. Very few medical errors result in litigation,^22^,^23^ and this analysis of closed-claims data found in the DSP provides only one perspective of the intricacies involved in clinical practice and medical negligence in emergency medicine.

## CONCLUSION

Of the 6,779 closed medical professional liability claims originating from ED or urgent care centers over a 15-year period, 65.9% were dropped, withdrawn, or dismissed; 22.8% settled for an average indemnity of $297,709; 7.6% went to trial; and 3.7% resolved by alternative dispute resolution/contract/unknown. In those that went to trial, juries returned verdicts for the defendant 92.6% of the time; however, claims where the jury returned verdicts for the plaintiff had the highest average indemnity of $816,909 of any claim resolution type. Acute myocardial infarction was the diagnosis with the highest ratio of paid-to-closed claims. Death was the most common outcome listed in closed claims; however, outcomes listing grave injury had more than double the average indemnity as paid claims listing death as the resulting injury ($686,239 vs $326,350).

## Figures and Tables

**Figure 1:**
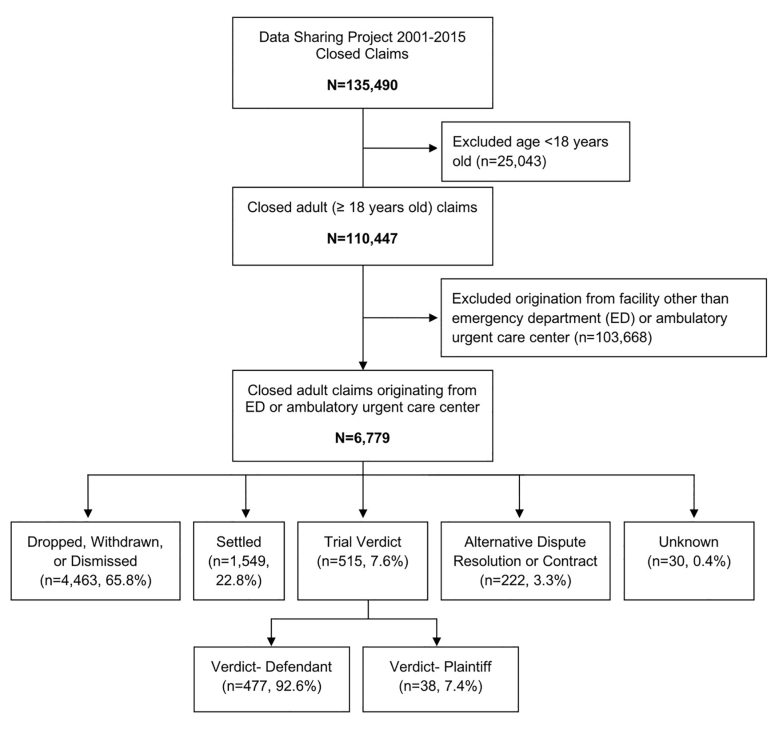
Claim resolution of closed claims in adult emergency departments or urgent care settings.

**Table 1:** Summary of claims submitted to the Data Sharing Project of the Medical Professional Liability Association during a 15-year period (2001–2015).

Description	Closed claims	Paid claims	% Paid-to-closed	Average Indemnity	Average defense expense	% of all closed claims	% of all paid claims
All claims for patients 18+ years	110,447	30,720	27.8	$308,083	$41,033	81.5%	85.2%
Emergency Department and Urgent Care Claims	6,779	1,799	26.5	$309,908	$41,047	5.0%	5.0%
Emergency Department	5,737	1,499	26.1	$321,034	$42,602	5.2%	4.2%
Urgent Care	1,042	300	28.8	$254,315	$32,484	0.9%	0.8%

**Table 2:** Resolution and outcomes of closed claims in adult emergency departments or urgent care settings.

Resolution	Closed claims	Paid claims	% Paid-to-closed	Average indemnity	Average defense expense	% of all closed claims	% of all paid claims
Dropped, withdrawn, or dismissed	4,463	-	-	$0	$25,996	65.9%	0.0%
Settled	1,549	1,549	100.0	$297,709	$55,260	22.8%	86.1%
Verdict-defendant	477	-	-	$0	$111,446	7.0%	0.0%
Verdict-plaintiff	38	38	100.0	$816,909	$159,716	0.6%	2.1%
Alternative dispute resolution/Contract	222	193	86.9	$279,380	$70,986	3.3%	10.7%
Unknown	30	19	63.3	$600,526	$55,001	0.4%	1.1%
TOTAL	6,779	1,799	26.5				

**Table 3:** Outcomes of the top five resulting medical conditions cited in closed claims in adult emergency departments or urgent care settings.

Top 5 resulting medical conditions	Closed claims	Paid claims	% Paid-to-closed	Average indemnity	Average defense expense	% of all closed claims	% of all paid claims
Cardiac or cardiorespiratory arrest	617	187	30.3	$340,622	$54,410	9.1%	10.4%
Myocardial infarction, acute	269	105	39.0	$306,487	$46,447	4.0%	5.8%
Aortic aneurysm	153	47	30.7	$369,872	$43,163	2.3%	2.6%
Pulmonary embolism	147	50	34.0	$302,996	$29,819	2.2%	2.8%
Appendicitis	134	39	29.1	$159,815	$28,432	2.0%	2.2%

**Table 4:** Outcomes based on top five chief medical factors cited in closed claims in adult emergency departments or urgent care settings.

Top 5 chief medical factors	Closed claims	Paid claims	% Paid-to-closed	Average Indemnity	Average defense expense	% of all closed claims	% of all paid claims
Errors in diagnosis	2,466	854	34.6	$338,362	$43,600	36.4%	47.5%
No medical misadventure	1,301	43	3.3	$294,140	$35,588	19.2%	2.4%
Improper performance	1,197	356	29.7	$289,941	$36,185	17.7%	19.8%
Failure to supervise or monitor case	352	119	33.8	$296,551	$41,761	5.2%	6.6%
Medication errors	232	58	25.0	$170,148	$33,834	3.4%	3.2%

**Table 5:** Outcomes according to severity of injury cited in closed claims in adult emergency departments or urgent care settings.

Severity of injury	Closed claims	Paid claims	% Paid-to-closed	Average indemnity	Average defense expense	% of all closed claims	% of all paid claims
Death	2,613	770	29.5	$326,350	$45,588	38.5%	42.8%
Grave injury	201	65	32.3	$686,239	$66,722	3.0%	3.6%
Major permanent injury	410	157	38.3	$505,965	$67,025	6.0%	8.7%
Significant permanent injury	617	162	26.3	$334,723	$47,168	9.1%	9.0%
Minor permanent injury	658	163	24.8	$248,662	$34,226	9.7%	9.1%
Major temporary injury	937	251	26.8	$215,244	$33,821	13.8%	14.0%
Minor temporary injury	1,027	188	18.3	$152,810	$27,376	15.1%	10.5%
Insignificant injury	179	27	15.1	$89,726	$14,914	2.6%	1.5%
